# Monthly Reports

**Published:** 1803-03-01

**Authors:** J. Ricards

**Affiliations:** No. 68, Hatton Street


### MONTHLY REPORTS.

*Cases admitted under the Care of the Surgeon of the Finsbury Dispensary, St. John's Square, Clerkenwell, from January 10, to February 10, 1803.*

| | | | |
|---|---|---|---|
| Phlegmone Artuum | 2 | Contusiones | 9 |
| ——— Testis | 1 | Spasma Maleoli | 5 |
| ——— Maxilla | 1 | Concussio Cerebri | 1 |
| Erysipelas | 1 | Combustura | 3 |
| Mastodynia | 1 | Vulnera | 2 |
| Abscessus Artuum | 3 | Lues | 5 |
| ——— Psoas | 1 | Gonorrhœa | 3 |
| ——— Lateris | 1 | Hydarthus Genu | 3 |
| ——— Cervicis | 1 | Hæmorrhois | 1 |
| ——— Ani | 2 | Stricturæ Urethra | 3 |
| Empyema | 1 | Tinea | 1 |
| Mortificatio | 1 | Prolapsus Uteri | 1 |
| Ulcera Artuum | 9 | Spina Incurvata | 1 |
| Pernio | 1 | Hydrocephalus | 1 |
| Leucoma | 2 | | |
| Fractura Costæ | 1 | Total | 68 |

It is a general remark among Surgeons, the truth of which is frequently evinced by observation, that in no injury or disease is there so much doubt, with respect to the prognosis, as in injuries of the head; it not very unfrequently happening, that injuries which in the first instance were apparently of little moment, and unattended by any symptoms creative of alarm, terminate fatally; while, on the contrary, a variety of cases might be brought forward, which, in the first infliction of the injury, from either the attendant symptoms or the local appearances, were productive of considerable fears, and yet eventually had a happy termination.

The above case (in the list) of concussion was in the first instance attended with all those symptoms which are usually considered as prognostic of imminent danger; but to plentiful bleeding, and other evacuations, these symptoms in a very short time yielded, and no ill effects from the injury were experienced.

That the structure of the brain may be very considerably injured, either from a wound or from compression by a fractured portion of bone, there are various instances to prove, without producing even a single symptom of deranged state of that organ. A few months since, a case occured to me in private practice, of a boy, who in consequence sequence of a kick of a horse, suffered a fracture of the cranium. On the first reception of the injury he became comatose, and had other symptoms of compression or concussion. A surgeon in the neighbourhood being called, bled him freely, and administered a cathartic medicine, and dressed the wound of his head. He in the course of an hour became sensible, and was then free from *symptoms*. After a few days he was brought home, when I first saw him, being requested to attend him on account of the wound of the scalp, as no suspicion had been entertained of any further injury. On examination I found the wound was in the forehead, and that there was a considerable fracture of the bone, which was wedged inward, having penetrated the membranes, as was evident from the discharge of a large portion of brain by the wound; yet although the injury to the cranium, to the brain and its membranes, was so great, yet no symptom existed of any affection of those organs: in consideration whereof, I did not perform the operation of the trephine; nor was it required, as the subsequent event proved; as he recovered perfectly without a single disagreeable symptom, or any unpleasant occurrence, except from the delay which was occasioned to the cicatrization of the scalp from the exfoliation of two or three small splinters of bone.

This case certainly leads to the practical question of, What are the cases that require the use of the trephine? On this point some of the first surgeons of the present day have differed: some advise the operation in every case of fracture of the bone with depression, whether attended with symptoms of injured brain or otherwise; while others never operate, except the presence of symptoms indicate the necessity.

What is our intention in performing this operation? Certainly, to remove pressure from the brain or its membranes, on which certain symptoms depend; or, if such symptoms have not arisen, to prevent them by an elevation of the offending cause. It therefore appears to me proper, that in the first instance of an accident of this kind, where the bone is much depressed, that pressure should be removed, without waiting for the accession of symptoms; for although, at first, symptoms of compression may not occur from the primary mechanical action of the bones, yet when inflammation arises, this action, if suffered to continue, may not only aggravate the inflammation, but may, in the then increased state of tension, prove a cause extremely productive of actual compression. Had I been therefore called, in the first instance, to this patient, patient, I should have certainly judged the operation advisable; but at the period when I first saw him, he had passed above a week without any symptoms of inflammation; there was a plentiful discharge of the brain likewise, which I concluded, in some measure, tended to prevent compression by means of the bone; I therefore thought, that in this state of things there was more fear of inflammation from an operation, from a more free exposure of the brain, and probably on disengaging the bone a still further laceration of the membranes, than by suffering the parts to remain at rest, and preventing internal compression by free evacuations and a strict antiphlogistic regimen.

The certainty to which the safety, mildness, and prophylactic effects of the Jennerian Inoculation is now reduced, has induced the Governors of this Institution to publish a solicitation to the Poor, to submit their families to this operation, and an order that all who choose, shall be inoculated, and properly attended to, without any letters of Recommendation. That the Order should be made still more public, I have annexed a Copy.

*No.* 68, *Hatton Street.* J. RICARDS.

---

FINSBURY DISPENSARY, St. John's Square, Clerkenwell.

THE power of the Cow-Pox as a preventive of the Small-pox seeming now fully established, the following well-authenticated facts are presented to the public, to encourage persons to embrace this favourable opportunity of avoiding the baneful effects of the Small-pox in their families, one of the most dreadful maladies that afflict the human race.

1. The Cow-pox, properly conducted, has not failed, in a single instance, of proving a certain preventive of the Small-pox.

2. The Cow-pox is a disease unattended by fever, and perfectly free from danger.

3. The Cow-pox is seldom attended with eruptions, and therefore cannot disfigure the skin.

4. As the Cow-pox is not infectious, it cannot be communicated in any other way than by inoculation: the mother, therefore, is not liable to disease from suckling her child.

5. The Cow-pox is never productive of other diseases, or any blemish whatsoever.

6. The Inoculation for the Cow-pox may be performed at any age, or under any state of the constitution; even in children during the time of teething.

Signed, JOHN REID, M. D.<br>THO. JAMESON, M. D. } Physicians to the Dispensary.<br>JASP. RICARDS, Surgeon to the Dispensary.<br>MICH. BARTLETT, Apothecary.

A parti-

A particular mark remaining upon the arm being necessary to render it effectual; patients are defired not to discontinue their attendance upon the Surgeon until he has given them a discharge.

The Governors of the Dispensary have therefore resolved, That all persons applying may be inoculated with the TRUE Vaccine Matter, and properly attended, free of expence, and without a letter of recommendation. Application must be made to Mr. Ricards, the surgeon, at the Dispensary, on Monday in every week, at twelve o'clock.

By Order. A. RHODES, Secretary.

*February* 16, 1803.

